# Practice Pointer: “My feelings are not on mute – ZOOM and Balint Practice Pointers”

**DOI:** 10.1111/ajr.12944

**Published:** 2022-11-04

**Authors:** Tamara Holmes

**Affiliations:** ^1^ Deakin University Elliminyt Victoria Australia

A case example:

*A doctor presented on the patient with chronic illness; who presents with a range of somatising illness. This included seeking pain management prescriptions, on‐going low mood and a long history of traumatic relationships. In the initial case presentation, the doctor voiced their frustration at not being able to bear the patient in the room, wishing they would leave and hoping they would not come back*.

Reading this case, I am sure patients immediately come to your mind and with it, the “heart‐sink”^1^ that comes with this thought. Balint work enables this to be shared in the group setting and lightens the load in our practice. Throughout the pandemic, GP's (and other clinicians) have been confronted with challenges which have battered at our resilience and ability to have boundaries after the working day is done. Traditionally, my practice has been face‐to‐face peer support using the Balint method. With restrictions in place, groups transitioned to ZOOM. While there were challenges to consider, the group endured and found new ways of reflecting on their feelings and attention to the doctor/patient relationship.

Balint work is a regular session/meeting among clinicians with the purpose of exploring the emotional content of their practice. The group allows for safe exploration of the challenging situations from clinical practice which the practitioner may find are on their mind long after the exit from work. Michael Balint was a psychoanalyst who began this work with his wife, Enid, in the 1950s to assist family physicians with the task of thinking about their patients in a psychological manner.^2^

The work of Balint, at the core, “is to make good health care professionals better health care professionals”.^3^ Over the course of the pandemic, Balint groups have been formed, and continued. They provide a reflective space not only for GP's, as the original participants in Balint work,^4^ but also for other clinicians with a view of holding the clinician–patient relationship in mind.

Often, we need to “mute” our responses and dampen the reaction to what's presented in the privacy of the room. Balint allows for the full expression of the range of our emotions and experience. The move to ZOOM, in this practice example, enabled the work to continue through a (hopefully), once‐in‐a‐lifetime pandemic. Coming from a regional area in Victoria, there were high case numbers of COVID‐19 throughout the region. The time carved out to really reflect, not be rushed and to provide peer acceptance and support was invaluable. All participants reported feeling lighter upon finishing group and more able to make clearer decisions in their day‐to‐day work.

Balint groups can enable a range of benefits to clinicians. The need for ways of engaging in clinical reflective practice has been amplified throughout the pandemic. Huang et al.^5^ found Balint groups mediated physician burnout. Balint groups may also increase empathy in the clinical relationship among medical students.^6^ Throughout the past 2 years, reflective practice groups have generally moved online. This has supported access and the on‐going consideration of enabling helping work to not overwhelm our limited emotional resources. Over this time, I have facilitated a regional GP group which transitioned to the ZOOM platform in March 2020. Nease et al.^7^ found through a small study, common outcomes of Balint work were still achieved through this platform.

The beauty of Balint lies in the ability of the group to explore the emotional realm of the doctor–patient relationship. Through a structured leadership process, the group thinks and feels what they imagine is happening for the doctor, the patient and in the relationship between them. The case presentation is usually prompted through consideration of what is causing perturbation, or worry. These are the cases which we carry as an additional load or wake us up in the dead of night. This structure continued in the online setting.

The benefits of moving online, enabled the group to continue to meet. The body language was used without the limitations of masks on inter‐personal interaction. The doctors highlighted the ease of logging on, doing the work and then not having to drive home which adds to an already long day. Other notable aspects, after meeting for 3 years in person previously, were all members found that the Balint work intimacy was not impacted by the change to becoming disembodied. In some ways, it allows for a more powerful personal reaction to the material which is presented without the awareness of the other physical bodies in the room. The focus can be on the internal content, the awareness of our own physical and emotional reactions to the material and the ability to really think and feel deeply to undertake the Balint work.

The challenges of the move to the on‐line environment included I.T. deficits. Being located regionally meant at times, verbal overtures were required to ensure members were present and thinking, rather than frozen out of the group. Another challenge was the decreased opportunity for informal collegial support which occurs pre and post group in a face‐to‐face setting. This was mediated by allowing 10–15 min at the beginning of the ZOOM group for a check‐in on each member prior to starting the Balint case discussion. The opportunities presented through the pandemic have taught us Balint groups are able to be successful via ZOOM which creates greater flexibility and access for GP and other health professionals.

Balint work is transferable to other types of health care professionals. I ran groups with social workers and students, art therapists and organisational consultants online. As a trainee leader on the pathway with the Australian and New Zealand Balint Society, I engage in my own Balint supervision with an accredited leader‐trainer and also participate in a Balint group. This immersion in the work allows me to move between the domains of meta‐cognition, thinking and feeling to ensure psychological safety exists in the group. This scaffolding allows for clear boundaries, attention to the Balint frame and ultimately ensures the emotional protection for group members.

## CONFLICT OF INTEREST

No conflict of interest.

## References

[1] Wilson H , Cunningham W . Being a doctor: understanding medical practice. Otago; 2017 [cited 2022 Feb 26]. Available from: https://search.ebscohost.com/login.aspx?direct=true&AuthType=sso&db=cat00097a&AN=deakin.b4676048&authtype=sso&custid=deakin&site=eds‐live&scope=site

[2] Salinsky J . Balint under the microscope: what really happens in Balint groups? Int J Psychiatry Med. 2018;53(1–2):7–14. 29226753 10.1177/0091217417745287

[3] Sternlieb JL . Demystifying Balint culture and its impact: an autoethnographic analysis. Int J Psychiatry Med. 2018;53(1–2):39–46. 29380650 10.1177/0091217417745290

[4] Balint M . The Doctor, his patient, and the illness. New York, NY: International Universities Press; 1957.

[5] Huang L , Cui H , Zhang X , Wu W , Harsh J , Wu J , et al. A randomized controlled trial of Balint groups to prevent burnout among residents in China. Front Psychiatry. 2019;10:957. 10.3389/fpsyt.2019.00957 32116808 PMC7026367

[6] Lemogne C , Buffel du Vaure C , Hoertel N , Catu‐Pinault A , Limosin F , Ghasarossian C , et al. Balint groups and narrative medicine compared to a control condition in promoting students' empathy. BMC Med Educ. 2020;20(1):1–8. 10.1186/s12909-020-02316-w PMC7654605 33167952

[7] Nease DE , Lichtenstein A , Pinho‐Costa L , Hoedebecke K . Balint 2.0: a virtual Balint group for doctors around the world. Int J Psychiatry Med. 2018;53(3):115–25. 29609525 10.1177/0091217418765036

